# Hepatitis E virus‐associated Guillain–Barre syndrome: Revision of the literature

**DOI:** 10.1002/brb3.1496

**Published:** 2019-12-11

**Authors:** Hang Liu, Ying Ma

**Affiliations:** ^1^ Department of Neurology Shengjing Hospital China Medical University Shenyang China

**Keywords:** antiganglioside antibodies, extrahepatic manifestations, Guillain–Barre syndrome, hepatitis E virus, infections, peripheral neuropathies, viral replication

## Abstract

**Introduction:**

The association between preceding infection of hepatitis E virus (HEV) and Guillain–Barre syndrome (GBS) has been found for more than a decade, while hepatitis E virus‐associated Guillain–Barre syndrome (HEV‐associated GBS) still remains poorly understood. Initially discovered in 2000, the association between GBS and HEV has been focused by neurologists increasingly. Five percent of patients with GBS had preceding acute HEV infection in the Netherlands and higher rate was found in Bangladesh (11%) where HEV is endemic.

**Method:**

An extensive review of relevant literature was undertaken.

**Results:**

Hepatitis E virus infection may induce GBS via direct viral damage according to recent research findings. On the other hand, the presence of antiganglioside GM1 or GM2 antibodies in serum of some HEV‐associated GBS patients indicates that HEV infection may trigger GBS by activating autoimmune response to destroy myelin or axon mistakenly. Management of HEV‐associated GBS has no obvious difference from GBS. It mainly consists of supportive therapy and immunotherapy. Intravenous immunoglobulin (IVIG) or plasma exchange (PLEX) was used in most reported cases, which is the main strategy for clinical treatment of HEV‐associated GBS. Whether antiviral therapy could be additional strategy other than the routine therapy to shorten the length of disease course is one of the most urgent problems and requires further study.

**Conclusions:**

An overview of possible pathogenesis will gain a first insight into why HEV, traditionally recognized as only hepatotropic, can induce many neurological disorders represented by GBS. Moreover, understanding of the underlying mechanisms may contribute to development of a novel therapeutic strategy. This review also summarizes management and clinical characteristics of HEV‐associated GBS, aiming to achieve early recognition and good recovery.

## Graphical abstract

In this review, we gain a first insight into the possible pathogenesis mechanisms and summarize current therapeutic strategies and clinical characteristics of hepatitis E virus‐associated Guillain–Barre syndrome. Meanwhile, we also emphasize the possibility of development of a novel therapy.

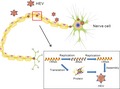

## 1 INTRODUCTION

**Figure 1:**
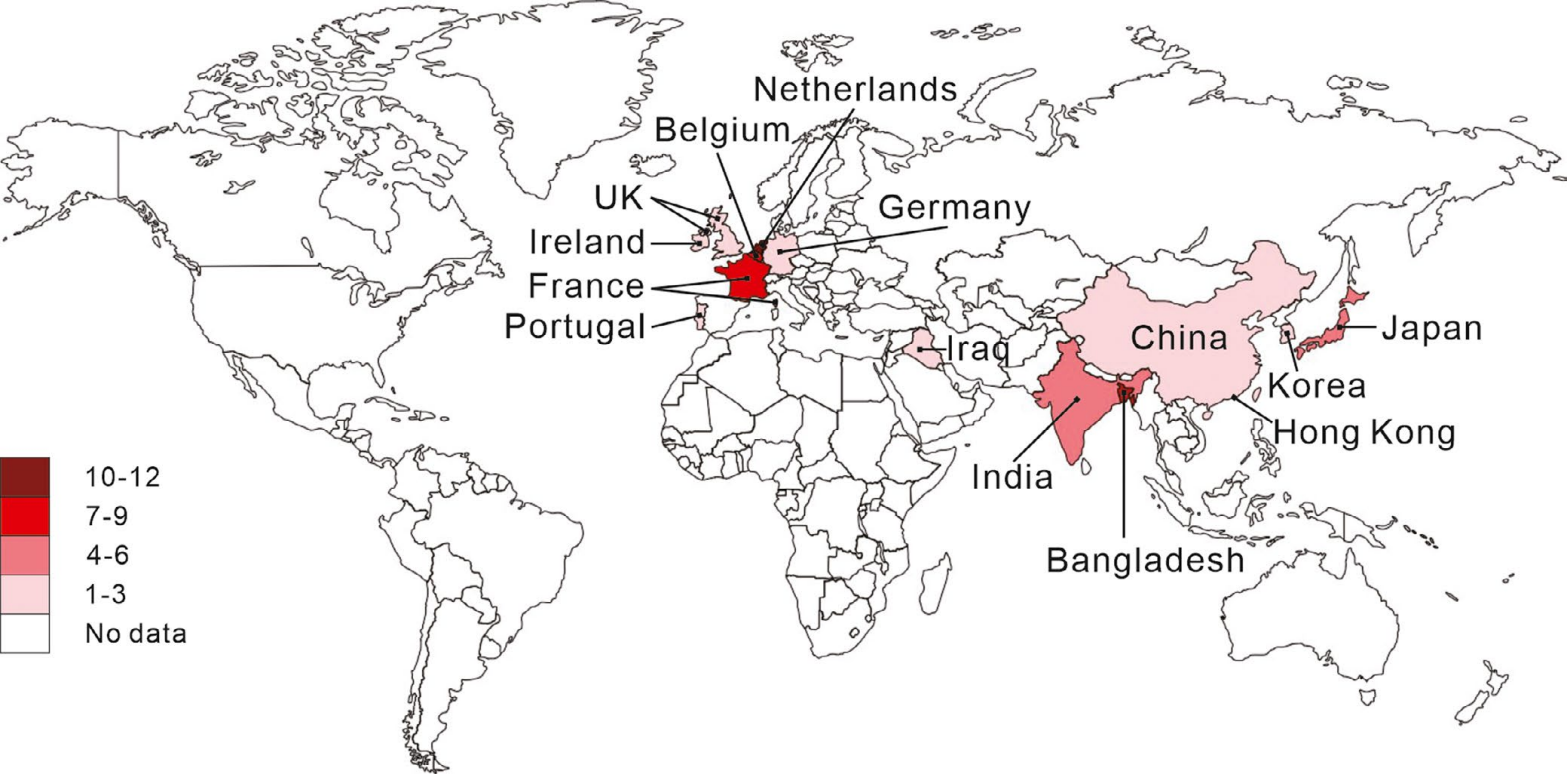
Geographic distribution of human cases of hepatitis E virus‐associated Guillain–Barre syndrome. From 2000 to 2018, 59 cases of hepatitis E virus‐associated Guillain–Barre syndrome have been reported worldwide, among which 58 have available information of country. Thirty‐eight cases have been reported in developed countries or regions in comparison with 20 cases in developing countries, probably due to higher diagnostic rate

Hepatitis E virus (HEV) infection is the main cause of hepatitis worldwide, which can be seen in developing country more commonly. HEV infection is usually acute and self‐limiting, while it may become chronic in immunocompromised individuals (Kamar, Dalton, Abravanel, & Izopet, 2014). There are 4 major genotypes of HEV (genotype 1 to 4; Lu, Li, & Hagedorn, 2006). Infection with HEV in human has two definitive epidemiological patterns. In developing country, HEV 1 and HEV 2 spread between humans by the fecal‐oral route, mostly via contaminated water. The feature of transmission explains frequent sporadic cases and occasionally large outbreaks in areas of poor sanitation. In developed countries, HEV 3 and HEV 4 spread from animal reservoirs to humans zoonotically (Hoofnagle, Nelson, & Purcell, 2012; Kamar et al., 2012; Purcell & Emerson, 2008; Teshale, Hu, & Holmberg, 2010), and recently the amount of sporadic HEV infection in developed country has been increased (Dalton, Webb, Norton, & Woolson, 2016), indicating that infection with HEV is getting more notable in developed country than before. A study among the U.S. born individuals has shown that the weighted seroprevalence of HEV (immunoglobulin G [IgG]/immunoglobulin M [IgM]) was increased from 4.5% in 2013–2014 to 8.1% in 2015–2016, and the seroprevalence of IgM indicating recent HEV infection has nearly doubled (Cangin, Focht, Harris, & Strunk, 2019). Many extrahepatic manifestations associated with HEV infection have been reported, of which neurological disorders primarily manifestating as Guillain–Barre syndrome (GBS) should be taken noticed by neurologists. Sood, Midha, and Sood (2000) firstly reported the case of GBS associated with HEV infection in India. Since then an increasing number of cases have been diagnosed in the last several years (Figure 1). The largest number of cases was reported from Bangladesh, followed by the Netherland. What is fascinating is that the total number in developed countries is no less than that in developing countries. This breaks the impression that HEV‐associated GBS commonly occurs in those unsanitary regions.

Guillain–Barre syndrome is a postinfectious and autoimmune‐induced peripheroneural disorder, characterized by a rapidly progressive bilateral and symmetric weakness of limbs in its classic form (acute inflammatory demyelinative polyradiculoneuropathy, AIDP). Although AIDP was more common in reported cases, any other types of GBS may follow HEV infection. About two‐thirds of patients have preceding infection within 3 weeks before onset of weakness (Stevens, Claeys, Poesen, Saegeman, & Van Damme, 2017). Some common infectious agents causing GBS are as follows: Campylobacter jejuni, cytomegalovirus (CMV), Epstein–Barr virus (EBV), Mycoplasma pneumoniae, Haemophilus influenzae, and hepatitis B virus (Hadden et al., 2001; Jacobs et al., 1998).

The purpose of this review is to clarify the pathogenesis of HEV‐associated GBS, the clinical presentations and diagnosis with a particular insight provided to the neurologists and hepatologists, and outline subsequent management and prevention. Although existing therapies are limited in providing a functional improvement, new programs of treatment should still be designed to employ in combination or sequential therapeutic strategies along with the scientific understanding of pathophysiological mechanisms of HEV‐associated GBS.

## 2 PATHOGENESIS

**Figure 2:**
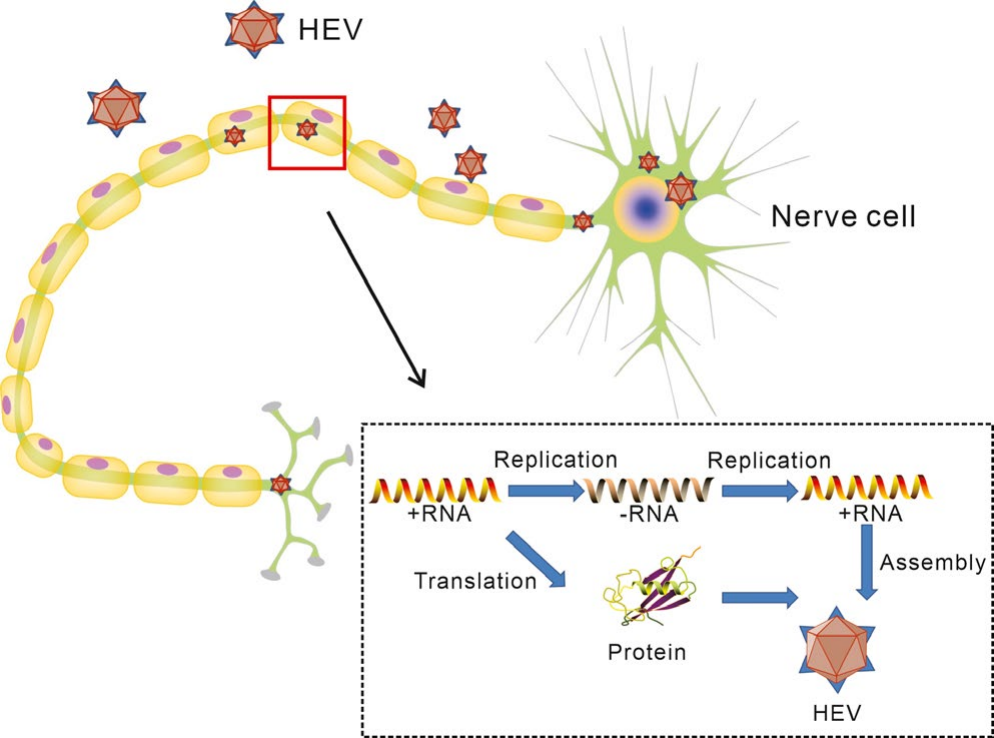
The possible mechanism in the context of hepatitis E virus (HEV) replication. HEV replication causes direct viral damage to peripheral nervous system. HEV can experience complete replication process in nerve cells

**Figure 3:**
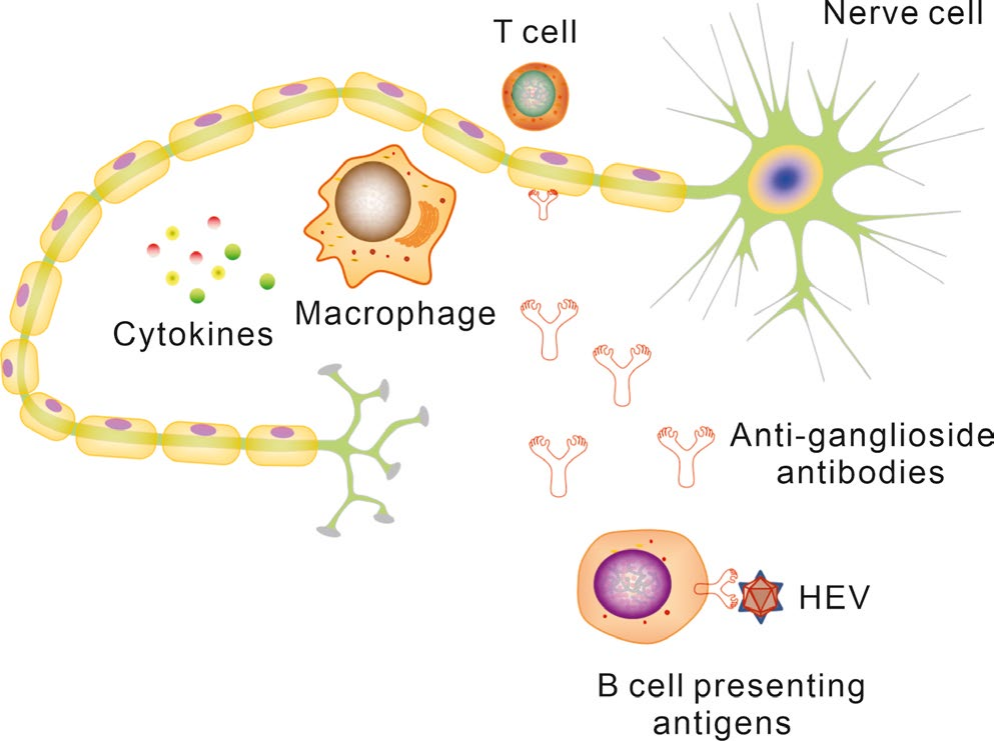
The possible mechanism in the context of indirect immune response. HEV infects human and triggers an immune response. Molecular mimicry between infectious agents and peripheral nerve self‐antigens may result in nerve injury

The clear mechanisms by which HEV can induce GBS are still unknown, but two possible pathogenesis causes have been proposed according to published studies. One is direct viral damage due to HEV replication in neurological system (Figure 2), and the other is indirect immune response, also called molecular mimicry (Figure 3).

Hepatitis E virus is seen to be only hepatotropic traditionally. But according to a recent research by Zhou et al., it has demonstrated that HEV can not only infect hepatic tissue but also infect neural cells directly in vitro. Furthermore, neuronal derived cell lines represented by glioblastoma cells have ability to support long‐term replication and the production of infectious HEV. For mice inoculated with HEV particles intravenously, viral RNA and protein were detected in brain tissue (Zhou et al., 2017). The result provides evidence for HEV having ability to infect neural tissue in vivo in animal models. In another study conducted by S.A. Drave et al., HEV was transfected into multiple human neuronal derived cell lines. Finally, all tested cell lines supported full‐length RNA replication, and viral capsid protein as a marker of assembly and release was detectable in different neuronal cell lines (Drave et al., 2016). Further assay shows that some cell lines have the ability to support HEV entry (Debing et al., 2014; Drave et al., 2016; Shukla et al., 2011). As known, blood–brain barrier (BBB) mainly consists of brain endothelial cells and astrocytes, and the tight junctions of them are crucial for maintaining integrity of BBB. It has been proved in animal models that HEV can break through BBB by sabotaging the relative junction complex and the endothelial cell structures that play an important role in preserving the integrity of BBB (Shi et al., 2016). In addition, HEV infection can reduce expression of zonula occludens‐1 (ZO‐1), a key tight junction protein presented between the cerebral endothelium and the astrocyte endfeet (Hamm et al., 2004; Liu, Wang, Zhang, Wei, & Li, 2012). HEV RNA has been detected in the cerebrospinal fluid (CSF) from some patients with HEV‐associated GBS (Comont et al., 2014; Troussière et al., 2018). These results show that HEV lead to GBS very likely by infecting peripheral nervous system directly. At the same time, HEV can disrupt BBB and release into CSF.

Another relatively well‐known hypothesis is that HEV induces GBS by autoimmune response with cross‐reactivity, also called molecular mimicry, which has been confirmed to explain another similar postinfectious GBS, Campylobacter jejuni‐associated GBS (Doorn, Ruts, & Jacobs, 2008). Myelin and axonal glycolipids are easy to be considered as antigen targets of antiganglioside antibodies especially in dorsal and ventral spinal roots and the sensory and motor nerve terminals, which are more freely exposed to circulating factors (Willison, 2018). This vulnerable characteristic of GBS‐affected sites can partly explain the autoimmune mechanism of acute infection‐induced GBS. Current view holds that when infectious organisms, especially those having the same epitopes with the host's peripheral nerves, invade into human, the host initiates immune response against the foreign infectious organisms and mistakenly attacks myelin or axon (Hughes & Cornblath, 2005). Antibodies produced by B cells originally aim to exotic pathogens will fight against autoantigens, mainly the ganglioside presenting in nerve cell membrane of myelin and axon, which can destroy the molecular topography of nodal and paranodal proteins and induce demyelination or axonal degeneration (Kaida et al., 2008). T cells and a variety of cytokines may be involved in this pathological process by activating endoneurial macrophages to release toxic nitric oxide radicals and assisting B cells to produce antibodies. It has been demonstrated that antiganglioside GM2 antibodies involve in the pathogenesis of CMV‐associated GBS and CMV‐infected fibroblasts express ganglioside‐like epitopes specifically recognizing anti‐GM2 antibodies (Ang et al., 2000; Yuki, 2001). Despite lack of in vitro and animal model studies directly demonstrating that HEV can stimulate immune system to produce antiganglioside antibodies, several cases of GBS triggered by HEV infection showed positive serum antiganglioside antibodies, suggesting possible molecular mimicry mechanism involving in the pathogenesis (Bandyopadhyay et al., 2015; Chen, Zhou, Zhou, Wang, & Tong, 2014; Comont et al., 2014; Cronin, McNicholas, Kavanagh, Reid, & O'Rourke, 2011; Fukae et al., 2016; Loly et al., 2009; Maurissen, Jeurissen, Strauven, Sprengers, & De Schepper, 2012). Furthermore, serum antiganglioside antibodies are generally IgG in GBS and clinical variants following acute infection, notably Campylobacter jejuni. However, in HEV‐associated GBS, serum antiganglioside antibodies are mostly IgM. It indicates that the molecular mimicry mechanism in GBS induced by HEV is not exactly the same as other pathogens. Moreover, the viral enzymes involved in genome replication, the viral capsid protein, and a phosphoprotein, are separately encoded by three open reading frames (ORFs) of HEV viral genome and these proteins have not been found structural similarity to peripheral nerve components (Tyler & Pastula, 2017). This possible relationship between antiganglioside antibodies and HEV‐associated GBS should be further studied both in vitro and vivo to confirm the mechanism.

## 3 CLINICAL PRESENTATION

We searched PubMed database to identify previously published case reports and clarify the clinical characteristics of HEV‐associated GBS from December 2000 through December 2018, using the keywords “Guillain‐Barre Syndrome” AND “hepatitis E.” Fifty nine cases describing HEV‐associated GBS were counted, and the clinical characteristics of these cases are summarized in Table 1. The mean age of the reported patients was 52 years (19–73 years), and 32 men were counted in a higher proportion. Most cases were found in Western Europe and Southern and Eastern parts of Asia. These patients developed HEV‐associated GBS after experiencing mild or moderate hepatitis‐like symptoms, including fever, nausea, malaise, anorexia, vomiting, abdominal pain, hepatomegaly, and jaundice in several days. The mean time of delay between acute hepatitis E and GBS symptoms was 12 days (with a range of 3–75 days). Neurological manifestations vary from different clinical subtypes acquired after delay, typically presented as numbness and weakness in the lower limbs rapidly progressing to quadriplegia with or without involvement of respiratory muscles or muscles innervated by the cranial nerves. Other more prevalent symptoms include the triad of oculomotor weakness, areflexia, and ataxia in Miller Fisher syndrome (MFS), pure paraparesis, pharyngeal–cervical–brachial weakness, bilateral facial palsy, bilateral lumbar polyradiculopathy, and acute severe midline back pain. The most meaningful physical findings are diminished or absent tendon reflexes, of which the severity is relevant to degree of limb weakness. Pathologic reflexes are usually negative in patients with HEV‐associated GBS.

**Table 1:** Clinical characteristics of hepatitis E virus‐associated Guillain–Barre syndrome

Reference	Country	No. of cases	Age, mean (*SD*) [range], years	Sex	Delay hepatitis GBS manifestation	Nerve conduction study	Antiglycoprotein antibody	IgM	HEV RNA	Treatment	Recovery/Delay
Sood et al. (2000)	India	1	50	M	5 days	AIDP	NT	+	NT	Supportive	Full/1 month
Kumar et al. (2002)	India	1	35	M	17 days	AMSAN	NT	+	NT	MV/IVIG	Full/2 weeks
Kamani et al. (2005)	India	1	58	F	9 days	NT	NT	+	NT	IVIG/PP	Full/12 days
Khanam, Faruq, Basunia, and Ahsan (2008)	Bangladesh	1	20	M	10 days	AIDP & AMSAN	NT	+	NT	MV	Full/12 days
Loly et al. (2009)	Belgium	1	66	M	Few days	AIDP	GM2 IgM+	+	NT	IVIG	Full/4 months
Cronin et al. (2011)	Ireland	1	40	M	Concomitant	AIDP	GM2 IgM+	+	NT	MV/IVIG/PP	Full/6 months
Kamar et al. (2011)	France	1	60	F	Concomitant	AIDP	NT	+	Serum+, CSF−	IVIG	Partial/18 months
Maurissen et al. (2012)	Belgium	1	51	F	Concomitant	AIDP	GM1 & GM2 IgM+	+	Serum+	IVIG	Full/1 week
Del Bello et al. (2012)	France	1	65	M	Concomitant	AIDP	NT	+	Serum+	MV/IVIG/Ribavirin	Partial/2 months
Tse, Cheung, Ho, and Chan (2012)	Hong Kong	1	60	F	3 days	AIDP	NT	+	NT	PP	Full/1 month
Santos et al. (2013)	Portugal	1	58	M	17 days	AIDP	NT	+	Serum+	MV/IVIG	Partial/2 months
Sharma, Nagpal, Bakki Sannegowda, and Prakash (2013)	India	1	27	M	40 days	AIDP	NT	+	NT	IVIG	Full/NM
Geurtsvankessel et al. (2013)	Bangladesh	11	NM	NM	NM	NM	NT	+	Serum+ (*n* = 1)	NM	NM
van den Berg, Eijk, et al. (2014)	Netherlands	10	54 (13) [32–69]	6M, 4F	Mean: 5 days	AIDP (*n* = 5) AMAN (*n* = 1) AMSAN (*n* = 1) Equivocal (*n* = 2) Inexcitable (*n* = 1)	NT	+	Serum+ (*n* = 3) CSF− (*n* = 10)	NM	NM
Chen et al. (2014)	China	1	64	M	5 days	AIDP	GM2 IgM+	+	NT	MV/IVIG	Full/12 months
Scharn et al. (2014)	Germany	1	50	M	7 days	AIDP & AMAN	–	+	Serum+, CSF−	IVIG	Partial/5 months
Woolson et al. (2014)	NM	1	42	M	NM	NM	NT	+	Serum+	NM	Full/3 months
Comont et al. (2014)	France	1	73	M	Concomitant	NM	GM1+	+	Serum+, CSF+	IVIG	Full/2 months
Bandyopadhyay et al. (2015)	Japan	1	43	F	14 days	AIDP & AMAN	GM1+	+	Serum+, CSF−	MV/IVIG	Partial/NM
Higuchi et al. (2015)	Japan	1	49	M	10 days	AIDP	–	+	Serum+	IVIG	Full/3 months
Perrin et al. (2015)	France	2	57 (3) [54–60]	1F, 1M	Few days	AIDP	NT	+	Serum+ (*n* = 1)	IVIG (*n* = 2)	Partial (*n* = 2)/5 weeks; 13 weeks
Fukae et al. (2016)	Japan	3	53 (4) [49–59]	3M	NM	AIDP (*n* = 2) MSF (*n* = 1)	GM1 IgM+ (*n* = 1) GQ1b IgG+ (*n* = 1) – (*n* = 1)	+	Serum+ (*n* = 1)	IVIG (*n* = 3)	Full (*n* = 2)/NM Partial (*n* = 1)/NM
Ji et al. (2016)	South Korea	1	58	M	75 days	NM	NT	+	NT	IVIG	Full/12 months
Lei, Tian, Luo, Chen, and Peng (2017)	China	1	30	M	Concomitant	AIDP	NT	+	NT	IVIG	Full/3 months
Stevens et al. (2017)	Belgium	6	61 (11) [41–75]	4M, 2F	NM	AIDP (*n* = 1) AMSAN (*n* = 1) Equivocal (*n* = 1) Demyelinating (*n* = 2)^b^ Sensory neuropathy (*n* = 1)	–	+	Serum+ (*n* = 2)	IVIG (*n* = 4) PP (*n* = 1) Supportive (*n* = 1)	Partial (*n* = 5)/3−6 months^a^ Death (*n* = 1)/1 month
Salim, Davidson, Li, Leach, and Heath (2017)	UK	1	59	M	NM	AMSAN	–	+	CSF+	NM	Partial/3 months
Oh et al. (2017)	Korea	2	NM	NM	NM	NM	NT	+	NT	NM	NM
Troussière et al. (2018)	France	1	60	M	10 days	Demyelinating	NT	+	Serum+, CSF+	IVIG	Full/4 months
Zheng, Yu, Xu, Gu, and Tang (2018)	China	1	58	M	11 days	AIDP	–	+	NT	IVIG	Full/6 months
Al‐Saffar and Al‐Fatly (2018)	Iraq	1	19	F	11 days	AMAN	NT	+	NT	Supportive	Full/35 days
Abravanel et al. (2018)	France	1	34	F	NM	AIDP	NT	+	–	IVIG	Full/3 months

Abbreviations: ‐, negative; +, positive; AIDP, acute inflammatory demyelinating polyneuropathy; AMAN, acute motor axonal neuropathy; AMSAN, acute motor‐sensory axonal neuropathy; CSF, cerebrospinal fluid; F, female; GBS, Guillain–Barre syndrome; HEV, hepatitis E virus; IVIG, intravenous immunoglobulin; M, male; MSF, Miller–Fisher syndrome; MV, mechanical ventilation; NM, not mentioned; NT, not tested; PP, plasmapheresis.

a Follow‐up performed after 3–6 months.

b Demyelinating features, but insufficient for criteria for acute inflammatory demyelinating polyneuropathy.

## 4 DIAGNOSIS

The diagnosis of HEV‐associated GBS can often be established based on clinical presentations, physical findings, and positive serologic results for anti‐HEV IgM. In addition, the presence of anti‐HEV IgG or positive results using reverse transcriptase polymerase chain reaction (RT‐PCR) for HEV in serum samples support a definite HEV infection. Serologic test and RT‐PCR for other pathogens should be performed to rule out the possibility of cross‐reactivity. In 59 cases reported, the positive rate for IgM serum was 100%. Among the 44 cases with available details of RNA test in serum or/and CSF, 19 out of 59 cases (43.18%) were positive. In a word, the definition of acute HEV infection is the presence of anti‐HEV IgM using enzyme‐linked immunosorbent assay (ELISA), with or without IgG, and supplemented by detection of HEV RNA in serum using RT‐PCR (van den Berg, Eijk, et al., 2014). Abnormal liver function often indicates HEV infection. The levels of total serum bilirubin and/or liver enzymes, mostly serum alanine transaminase (ALT) and aspartate transaminase (AST), have a remarkable elevation, far beyond the normal reference value. The progression presents as monophasic illness pattern, where interval between onset and nadir of weakness varies from 12 hr to 28 days followed by subsequent clinical plateau follows. In addition, it is necessary to exclude other identified alternative diagnosis for weakness. Nerve conduction studies can be helpful in clinical practice, of which the electrophysiological findings are consistent with GBS. AIDP is the most common type featured as decreased motor nerve conduction velocity, prolonged distal motor latency, increased F‐wave latency, conduction blocks, and temporal dispersion. Other variants of GBS can be also observed, including acute motor axonal neuropathy (AMAN), acute motor–sensory axonal neuropathy (AMSAN), MFS, and sensory neuropathy. Lumbar puncture also plays a crucial role in improving diagnostic certainty. CSF analysis of GBS is characterized by albuminocytologic dissociation (elevation of CSF protein levels above laboratory normal value and CSF total white blood cell count <50 cells/μl). Besides, the CSF sample should be tested for HEV RNA. Positive results provide direct evidence that HEV invaded into nervous system and triggered GBS. Several cases have reported positive results (Comont et al., 2014; Troussière et al., 2018), demonstrating that searching for HEV RNA in CSF is important for improving diagnostic accuracy. For those patients with HEV exposure history (e.g., ingestion of raw meat, travel abroad, blood transfusion, contact with affected animals, or contaminated water), neurologists also should be on the alert.

## 5 MANAGEMENT

Currently, there is no evidence that HEV‐associated GBS has different responses to standard therapy for GBS or has specific prognosis (Tyler & Pastula, 2017). Patients with pure HEV infection do not require special treatment normally due to its spontaneous remission (Dalton, Webb, et al., 2016). However, GBS secondary to HEV infection is a potentially fatal disease and requires close attention to both general medical care and immunotherapy (Esposito & Longo, 2017; Willison, Jacobs, & Doorn, 2016). These supportive measures include monitoring respiratory function, mechanical ventilation or intubation, monitoring heart and hemodynamics, prevention of deep vein thrombosis, management of possible bladder and bowel dysfunction, management of neuropathic pain, early initiation of physiotherapy and rehabilitation, and psychosocial support. Immunotherapy with either intravenous immunoglobulin (IVIG) or plasma exchange (PLEX) has been proved the efficacy (French Cooperative Group on Plasma Exchange in Guillain‐Barré Syndrome, 1987; Hughes & Cornblath, 2005) and has been shown to be equally efficacious in the management of GBS (Hughes, 1997; Hughes, Swan, & Doorn, 2012; Hughes et al., 2007; Meché & Schmitz, 1992; The Guillain‐Barré syndrome Study Group, 1985). A clinical improvement following IVIG administration was proven to be statistically significant in a retrospective study. However, combined therapy of PLEX and IVIG was not effective when PLEX, the first‐line treatment, did not improve clinical outcome. The same is true for administration of PLEX following IVIG failure (Shalem, Shemer, Shovman, Shoenfeld, & Kivity, 2018). New antiviral therapy has been tried to apply in treatment programs for HEV‐associated GBS in the way of monotherapy or combination of ribavirin and immunotherapy (Del Bello, Arné‐Bes, Lavayssière, & Kamar, 2012). Among the 59 cases reported in the literature, 34 of which provided details in therapy and outcome. Twenty‐eight patients used IVIG, 4 used PLEX, 1 used ribavirin, and 7 used mechanical ventilation due to the involvement of respiratory muscles. Most of these patients had full neurological recovery after a period of time. Some patients without IVIG or PLEX still had spontaneous recovery. 

Some patients with HEV‐associated GBS developed respiratory failure resulting from involvement of phrenic nerve, in need of mechanical ventilation or intubation (van den Berg, Walgaard, et al., 2014; Esposito & Longo, 2017; Willison et al., 2016). The main predictors of respiratory insufficiency and mechanical ventilation have been fully clarified previously (Walgaard et al., 2010). Meticulous attention also should be paid to corresponding management and prophylaxis of other severe complications.

The effects of immunotherapy have been demonstrated by several randomized controlled trials (RCTs) in the past few decades (Hughes et al., 2014; Raphaël, Chevret, Hughes, & Annane, 2002). IVIG or PLEX should be started early in case of irreversible nerve damage. PLEX as the first line of therapy, the most effective when started within a week of symptom onset, can remove antibodies and complement, and improve T‐cell suppressor function (Wijdicks & Klein, 2017). The component of replacement fluid is usually 5% albumin or a crystalloid–colloid combination. PLEX can achieve earlier improvement of weakness and faster recovery, and lower the possibility of MV for HEV‐associated GBS. However, PLEX causes several adverse reactions, such as hypotension, hypocalcemia, and thrombocytopenia occasionally (Ansar & Valadi, 2015).

Intravenous immunoglobulin is considered to be as effective as PLEX for GBS and is generally accepted by the whole world based on a large number of trials. The pharmacological mechanism is probably neutralization of antibodies, blockade of Fc receptor, or immunomodulation on B cells and T cells. A common dosing schedule for IVIG is 0.4 g/kg/day at approximately 1–3 ml/min for 5 days (Wijdicks & Klein, 2017). IVIG has replaced PLEX as the optimal option as it has a lot of advantages, such as fewer side effects, widespread availability, peripheral intravenous access, and convenient time (Donofrio, 2017). The side effects of IVIG consist mainly of headache, fever, nausea, tachycardia, chest pain, and hypotension. However, fortunately, IVIG does not cause lasting damage to health. The cost of IVIG is higher than that of PLEX, which brings heavy burden for patients with GBS. A number of studies show that combination of PLEX and IVIG does not have additional benefit compared with either treatment alone (Hughes, 1997; Plasma Exchange/Sandoglobulin Guillain‐Barré Syndrome Trial Group, 1997). For patients who encountered a failure of the first‐line treatment, either IVIG or PLEX was not effective.

Oral and intravenous corticosteroids are not effective in the treatment of GBS (Hughes, Brassington, Gunn, & Doorn, 2016). In addition, combining IVIG with methylprednisolone also has been tried with some additional short‐term benefit. The combination may accelerate recovery due to correction for known prognostic factors, but has no impact on long‐term outcome and neuropathic pain (Koningsveld et al., 2004). Therefore, corticosteroids are not recommended as a standard therapy for GBS.

Hepatitis E virus‐associated GBS can lead to severe weakness, unbearable pain, malaise, prolonged course of disease, or incomplete recovery in some patients. Others might experience progressive neurological injury or a relapse even though standard immunotherapy was given. Therefore, greater efforts should be made to explore better treatment to improve the outcome of HEV‐associated GBS. If HEV results in GBS by the mechanism of direct viral damage, early intervention with antiviral drugs will play a key role in obtaining better prognosis (Woolson et al., 2014). It is worthy to be considered whether to add ribavirin to routine therapeutic methods especially for patients with positive HEV RNA in the blood or CSF (Dalton, Kamar, et al., 2016). Antiviral monotherapy or combination of ribavirin and immunotherapy may become a novel therapeutic strategy for treatment of HEV‐associated GBS. However, the efficacy of the treatment is uncertain at present due to the lack of large randomized controlled trials (RCTs). But on the other hand, antiviral drugs may trigger release of viral antigens and enhancement of immune response, thus bringing the risk of viral damage to neurological system or aggravating neurological injury. Immunocompetent patients normally clear the virus spontaneously after acute HEV infection, so it is not definitely necessary to give antiviral treatment for patients with HEV‐associated GBS if they have healthy immune system. Antiviral therapy might be taken into account once the patients acquire extremely serious HEV infection. Besides, for patients with chronic HEV infection or immunocompromised populations, that is, organ‐transplant recipients, it might be beneficial to use ribavirin to assist the poor immune system to remove viral organisms. Clearance of viral particles could lead to rapid recovery. Doctors should take the first step to lower the dose of immunosuppressors for organ‐transplant recipients if possible, which would be able to clear about 30% virus (Kamar, Izopet, et al., 2014). If virus is not cleared successfully or the method is impossible in practice, ribavirin therapy as an alternative method should be considered. Further studies are required to confirm the efficacy of ribavirin for HEV‐associated GBS. It is challenging and meaningful to find better treatments.

## 6 PREVENTION

The best strategy to prevent HEV‐associated GBS in developing countries is to build adequate sanitation, which can decrease the rate of HEV infection markedly. For developed countries, the prevention is problematic due to a number of possible infection routes, which might include thoroughly cooking of meat, vaccination of farmed pigs, and screening of blood donors. Human HEV vaccine has been proved to be safe and effective, but it is only available in China. More research should be performed to verify its immunogenicity and safety aiming at other countries. The vaccine is likely to become one of the most effective methods in the high risk groups with HEV infection. Early diagnosis and treatment is important for patients with HEV infection, in case of the attack of following GBS.

## 7 PROGNOSIS

Most patients with HEV‐associated GBS have favorable prognosis. Among the 36 cases with available details of recovery, 21 cases (58.3%) had experienced complete neurological recovery within several weeks to several months (Table 1). Some patients do not regain full strength in the movement of limbs. However, one patient died after cardiac arrest 1 month after the onset of neurological symptoms. Some features of HEV‐associated GBS might indicate a poor prognosis, including late age of onset, the need for intubation or MV within the first week of illness, and severe weakness (van den Berg, Walgaard, et al., 2014; Esposito & Longo, 2017; Willison et al., 2016). The recurrence rate and mortality are still unknown so far.

## 8 CONCLUSION

Hepatitis E virus infection was frequently associated with GBS or variants of GBS. Two possible pathogenesis mechanisms have been proposed and require further study to explore more details. One novel point of view is raised that HEV can induce GBS by damaging neurological system directly by means of viral replication, versus with traditional indirect immune response mechanism. Although PLEX or IVIG has been widely used to treat HEV‐associated GBS, it is worth discussing whether antiviral monotherapy or combination of ribavirin and immunotherapy can be used as a novel treatment. If the pathogenesis was clarified sufficiently, the answer to the question would be straightforward because direct neural infection could respond well to antiviral therapy. Prevention and early diagnosis of HEV‐associated GBS can be difficult and challenging because prodromal symptoms of infection are usually asymptomatic or mildly symptomatic. For physicians, it is particularly important to aware of this underlying trigger of GBS in their workup.

## CONFLICT OF INTEREST

The authors declare no financial or other conflict of interests.

## Data Availability

Data sharing is not applicable to this article as no new data were created or analyzed in this study.
